# Synthesis and Anti-Inflammatory Activities of Phloroglucinol-Based Derivatives

**DOI:** 10.3390/molecules23123232

**Published:** 2018-12-07

**Authors:** Ning Li, Shabana I. Khan, Shi Qiu, Xing-Cong Li

**Affiliations:** 1 Nation Center for Natural Products Research, Research Institute of Pharmaceutical Sciences, School of Pharmacy, The University of Mississippi, University, MS 38677, USA; ahmulining@163.com (N.L.); skhan@olemiss.edu (S.I.K.); sqiu@olemiss.edu (S.Q.); 2 School of Pharmacy, Anhui Medical University, Hefei 230032, China; 3 Department of Biomolecular Sciences, School of Pharmacy, The University of Mississippi, University, MS 38677, USA

**Keywords:** phlorogluciniol, acylphloroglucinol, anti-inflammatory, iNOS, NF-κB

## Abstract

The natural product phloroglucinol-based derivatives representing monoacyl-, diacyl-, dimeric acyl-, alkylated monoacyl-, and the nitrogen-containing alkylated monoacylphloro- glucinols were synthesized and evaluated for inhibitory activities against the inflammatory mediators such as inducible nitric oxide synthase (iNOS) and nuclear factor kappaB (NF-κB). The diacylphloroglucinol compound **2** and the alkylated acylphloroglucinol compound **4** inhibited iNOS with IC_50_ values of 19.0 and 19.5 µM, respectively, and NF-κB with IC_50_ values of 34.0 and 37.5 µM, respectively. These compounds may serve as leads for the synthesis of more potent anti-inflammatory compounds for future drug discovery.

## 1. Introduction

The natural product phloroglucinol (**1**) and its derivatives have found a wide range of applications as pharmaceuticals, cosmetics, textiles, paints, and dyes due to their diverse biological activities [1,2]. More than 700 naturally occurring phloroglucinol derivatives have been reported, of which acylphloroglucinol derivatives comprise the largest group [1,3]. Structurally fascinating acylphloroglucinols possess antidepressant, antimicrobial, antiviral, antitumor, antioxidant, and anti-inflammatory activities [4,5,6,7]. The synthetic antispasmodic drug flopropione (3-propionyl- phloroglucinol) is a representative of this chemical class [8,9]. 

**Figure 1:**
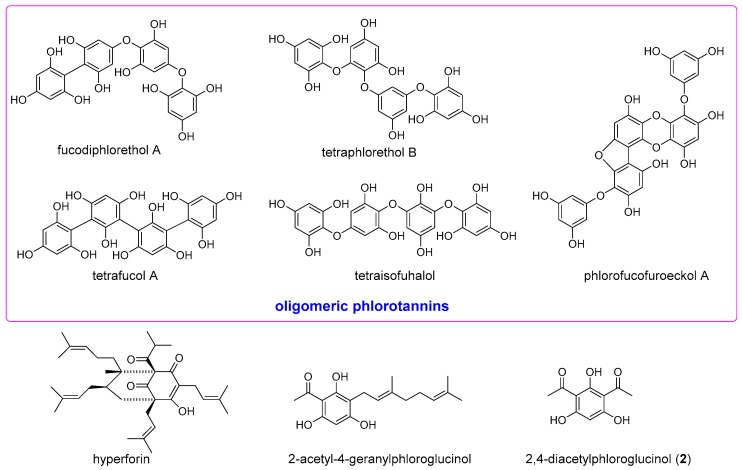
Representative phloroglucinol derivatives with anti-inflammatory activities.

The anti-inflammatory activity of the naturally occurring phloroglucinol derivatives acting on diverse molecular targets has drawn our particular attention. For example, complex phlorotannins from edible brown algae showed strong inhibitory effects on nitric oxide (NO) production in lipopolysaccharide (LPS)-induced RAW 264.7 macrophage cells [10]. Purified oligomeric phlorotannins containing fucodiphlorethol A, tetraphlorethol B, tetrafucol A, tetraisofuhalol, and phlorofucofuroeckol A (Figure 1) also demonstrated the ability to inhibit NO production and suppressing iNOS and cyclooxygenase (COX)-2 [11]. The acylphloroglucinol hyperforin (Figure 1) from St. John’s wort suppressed prostaglandin E_2_ formation in vitro and in vivo through inhibition of microsomal PGE_2_ synthase-1 [12]. 2-Acetyl-4-geranylphloroglucinol (Figure 1) isolated from the Rutaceaous plant *Melicope ptelefolia* is a strong dual inhibitor of 5-lipoxygenase (LOX) and COX-2 [13]. 2,4-Diacetylphloroglucinol (**2**) (Figure 1), a microbial metabolite of *Pseudomonas aeruginosa*, exhibited antimetastatic activity by mediating inhibition of reactive oxygen species (ROS), nuclear factor kappaB (NF-κB), B-cell lymphoma 2 (Bcl-2), matrix metalloproteinase-2 (MMP-2), vascular endothelial growth factor (VEGF) and primary inflammatory mediators such as tumor necrosis factor (TNF)-α, interleukin (IL)-6, IL-1β and NO [14]. In an effort to identify synthetically available phloroglucinol-based anti-inflammatory compounds, we synthesized several acylphloroglucinols and evaluated their iNOS and NF-κB inhibitory activities. 

## 2. Results and Discussion

**Scheme 1:**
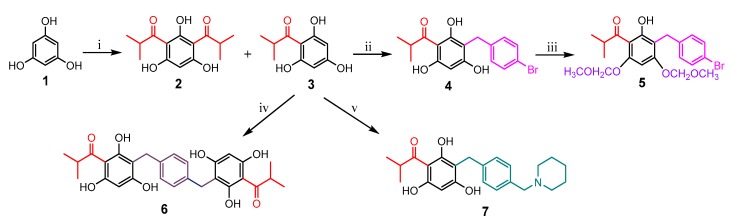
Synthesis of compounds **2**–**7**. Reagents and conditions: (i) nitrobenzene, AlCl_3_, rt for 0.5 h, then isobutyryl chloride, 65 °C for 21 h; (ii) NaOH, H_2_O, TBAI, 1-bromo-4-(bromomethyl)benzene, hexane, rt for 48 h; (iii) acetone, K_2_CO_3_, CH_3_OCH_2_Cl, rt for 24 h; (iv) NaOH, H_2_O, TBAI, *p*-xylylene dibromide, toluene, rt for 6 h; (v) piperidine, CH_2_Cl_2_, K_2_CO_3_, *p*-xylylene dibromide, NaOH, H_2_O, MeOH, rt for 7 h.

Based on the concept that both natural and synthetic acylphloroglucinols possess potent biological activities [6,7,13,14,15,16,17], seven compounds representing monoacyl-, diacyl-, dimeric acyl-, alkylated monoacyl-, and the nitrogen-containing alkylated monoacylphloroglucinols were synthesized (Scheme 1). Diacylphloroglucinol (**2**) and monoacylphloroglucinol (**3**) were prepared first according to the procedures described previously [18,19,20]. Using **3** as a starting material, compounds **4** and **6** were synthesized, respectively, in a one-step reaction adapted from a previously reported procedure [17]. Blocking the two non-hydrogen bonding hydroxyl groups in **4** using MOMCl afforded compound **5**. This synthesis was intended to assess the role of the hydroxyl groups on the phloroglucinol ring for activity. The nitrogen-containing compound **7** was designed to alter chemical nature of the target molecule based on previous reports [21,22,23]. It was synthesized by reacting **3** with 1-(4-(bromomethyl)benzyl)-piperidine that was prepared from commercially available piperidine and *p*-xylylene dibromide in the presence of CH_2_Cl_2_ and K_2_CO_3_. As can be seen from Scheme 1, the reaction conditions for the synthesis of these compounds were mild as all the reactions were conducted at room temperature. 

The identification of all the synthetic compounds was achieved by interpretation of NMR and MS data. Compounds **4**–**7** have not been reported prior to this study. It was noted that compound **7** produced an unusual ^1^H-NMR spectrum in CD_3_OD, displaying two sets of ^1^H-NMR signals in a ratio of approximately 1.5:1 associated with the *para*-substituted phenyl ring and some protons of the piperidine ring (Supplementary Materials). However, when the compound was measured in CDCl_3_ (with poor solubility), a single set of ^1^H-NMR signals appeared. Use of the weakly basic NMR solvent C_5_D_5_N afforded a ^1^H-NMR spectrum showing two sets of signals in a ratio of approximately 4:1 for the aromatic protons of the phenyl ring and the methylene attached to the piperidine ring. This interesting phenomenon can be explained by the fact that compound **7** is, to some extent, a zwitterionic molecule due to the presence of the acidic phenolic hydroxyl group on the phloroglucinol core and the basic amine functionality on the side chain. While CD_3_OD can strongly induce the protonation/deuteriation of the nitrogen atom, C_5_D_5_N has the ability to facilitate the formulation of a zwitterion, thereby resulting in the presence of an additional set of NMR signals. The ^1^H- and ^13^C-NMR signals of **7** in C_5_D_5_N were unequivocally assigned using 2D NMR of ^1^H-^1^H correlation spectroscopy (COSY), heteronuclear single quantum coherence (HSQC) and hetero- nuclear multiple bond correlation (HMBC) experiments.

In vitro anti-inflammatory testing of compounds **2**–**7** was conducted by assessing the inhibition of iNOS in LPS-induced macrophages (RAW 264.7) and inhibition of NF-κB in phorbol 12-myristate 13-acetate (PMA)-induced human chondrosarcoma cells (SW1353). As shown in Table 1, diacylphloroglucinol compound **2** showed the best activity, inhibiting iNOS and NF-κB with IC_50_ values of 19.0 and 34.0 µM, respectively, compared with the positive control parthenolide with IC_50_ values of 2.5 and 20.5 µM, respectively. The activity of alkylated acylphloroglucinol **4** against these two targets (IC_50_ 19.5 and 37.5 µM, respectively) was also very similar to compound **2**. When the hydroxyl groups in **4** were protected leading to compound **5**, iNOS inhibitory activity was decreased (IC_50_, 50 µM) while NF-κB activity was slightly increased (IC_50_, 29.0 µM). This suggests these two hydroxyl groups play a role for the activity and the differential activity between the two compounds may be associated with molecular lipophilicity. Dimeric acylphloroglucinol **6** inhibited iNOS to a similar extent (IC_50_, 19.0 µM) but inhibition of NF-κB was weaker (IC_50_, 85.0 µM). The nitrogen- containing compound **7** was only moderately inhibiting iNOS with an IC_50_ value of 49.0 µM, indicating the introduction of the amino group in this chemotype is not helpful. On the other hand, monoacylphloroglucinol **3** was only marginally inhibiting NF-κB with an IC_50_ value of 90.0 µM, indicating that additional acylation or alkylation on the phloroglucinol ring is necessary to enhance anti-inflammatory activity. Cytotoxicity testing using African monkey kidney fibroblast cell line (Vero) showed that compounds **2**–**7** were not cytotoxic up to a concentration of 50 µM

The anti-inflammatory activity observed for compound **2** against iNOS and NF-κB appears to be consistent with the previously reported data for the analogue 2,4-diacetylphloroglucinol [13]. Compound **2** was previously reported to possess potent in vitro antifungal activity against *Cryptococcus neoformans* [17]. An enhanced inflammatory cell response in *C. neoformans* infection has been observed via regulation of IL-23 and IL-12 [24]. Our findings suggest that the anti-inflammatory activity of compound **2** targeting iNOS and NF-κB would have a positive impact on its antifungal activity.

In conclusion, the natural product phloroglucinol-derived compounds were synthesized and evaluated for anti-inflammatory activity against iNOS and NF-κB as well as cytotoxicity against the mammalian Vero cells. This exploratory study has generated meaningful structure activity relationship information from the limited number of synthetic compounds. The diacylphloroglucinol compound **2** and the alkylated monoacylphloroglucinol compound **4** are dual inhibitors of iNOS and NF-κB. Further synthesis of these two chemotypes of compounds may afford compounds with improved anti-inflammatory activities which could be better candidates for future drug discovery.

## 3. Materials and Methods

### 3.1. General Experimental Procedures

The nuclear magnetic resonance (NMR) spectra using standard pulse programs were recorded at room temperature on a BrukerAvance DPX-400 spectrometer (Bruker, Billerica, MA, USA) operating at 400 (^1^H) and 100 (^13^C) MHz. The chemical shift (δ, ppm) values were calibrated using the residual NMR solvent and coupling constant (*J*) was reported in Herts (Hz). High-resolution time-of-flight mass spectrometry (TOF-MS) and electrospray ionisation mass spectrometry (ESI-MS) were measured on an Agilent series 1100 SL spectrometer (Agilent Technologies, Santa Clara, CA, USA) equipped with an ESI source. Thin layer chromatography (TLC) was performed on silica gel aluminum sheets (silica gel 60 F254, Merck, Darmstadt, Germany) and visualized by UV 254 nm and spraying 10% H_2_SO_4_ followed by heating. Column chromatography was done on normal-phase silica gel (230 × 400 mesh, J. T. Baker, Center Valley, PA, USA) and Sephadex LH-20 (75 μm, GE Healthcare Bio-Sciences AB, Uppsala, Sweden). Reactants and reagents including anhydrous phloroglucinol, nitrobenzene, aluminum chloride (AlCl_3_), *n*-butyl ammonium iodide (TBAI), 1-bromo-4-bromomethyl-benzene, chloromethoxymethane (CH_3_OCH_2_Cl), toluene, and piperidine were purchased from Sigma-Aldrich (St. Louis, MO, USA) in appropriate grades and were used without further purification. The yield of each synthetic product after column chromatography is reported. The ^1^H- and ^13^C-NMR assignments for all synthetic products and key intermediates with appropriate numbering systems are shown in the Supplementary Materials. Compounds **2** and **3** were synthesized according to the protocols described previously [18,19,20] and their identification was made by comparison of their NMR spectroscopic data (see Supplementary Materials) with those reported in the literature [17].

### 3.2. Synthesis of Compound ***4***

To a solution of compound **3** (3.0 g), NaOH (1.01 g), and TBAI (catalyzer, 500 mg) in H_2_O (225 mL) was added 1-bromo-4-(bromomethyl)benzene (2.5 g) in hexane (20 mL) dropwise. The mixture was stirred at room temperature for 48 h. The precipitated crude product was collected by filtration. The residue was purified by chromatography on silica gel using acetone:hexane (1:2) to afford **4** (1.67 g, yield 30.0%).

*1-[3-(4-Bromo-benzyl)-2,4,6-trihydroxy-phenyl]-2-methyl-propan-1-one* (**4**). ^1^H-NMR (400 MHz, CD_3_COCD_3_) δ: 14.33 (s, OH), 7.35 (2H, d, *J* = 8.2 Hz, H-14,14′), 7.24 (2H, d, *J* = 8.2 Hz, H-13, 13′), 6.16 (1H, s, H-4), 3.99 (1H, m, H-8), 3.85 (2H, s, H-11), 1.13 (6H, d, *J* = 6.6 Hz, H-9, 10). ^13^C-NMR (100 MHz, CD_3_COCD_3_) δ: 211.0 (C-7), 165.7 (C-1), 162.8 (C-5), 160.7 (C-3), 142.4 (C-12), 131.6 (C-14, 14′), 131.5 (C-13, 13′), 119.4 (C-15), 107.3 (C-6), 104.2 (C-2), 95.2 (C-4), 39.5 (C-8), 28.0 (C-11), 19.6 (C-9, 10). MS ESI-(−) *m*/*z* = 363.0/365.2 [M − H]^−^ for C_17_H_17_BrO_4_.

### 3.3. Synthesis of Compound ***5***

A suspension of compound **4** (346 mg) and K_2_CO_3_ (2.76 g) in acetone (35 mL) was stirred for 30 min, then CH_3_OCH_2_Cl (480 mg) was added. After stirring at room temperature for 48 h, the reaction mixture was filtered. The filtrate was concentrated, and the residue was purified by chromatography on silica gel with acetone:hexane (1:12) to give **5** (239 mg, yield 52.8%).

*1-[3-(4-Bromo-benzyl)-2-hydroxy-4,6-bis-methoxymethoxy-phenyl]-2-methyl-propan-1-one* (**5**). ^1^H-NMR (400 MHz, CDCl_3_) δ: 14.0 (s, OH), 7.35 (2H, d, *J* = 8.4 Hz, H-14, 14′), 7.21 (2H, d, *J* = 8.4 Hz, H-13, 13′), 6.46 (1H, s, H-4), 5.28 (2H, s, -OCH_2_), 5.24 (1H, s, -OCH_2_), 3.93 (2H, s, H-11), 3.82 (1H, m, H-8), 3.55 (3H, s, -OCH_3_), 3.41 (3H, s, -OCH_3_), 1.21 (6H, d, *J* = 6.7 Hz, H-9, 10). ^13^C-NMR (100 MHz, CDCl_3_) δ: 210.7 (C-7), 164.0 (C-5), 160.5 (C-1), 158.9 (C-3), 140.6 (C-12), 131.0 (C-14, 14′), 130.4 (C-13, 13′), 119.2 (C-15), 110.7 (C-2), 106.0 (C-4), 94.9 (-OCH_2_), 94.0 (-OCH_2_), 91.4 (C-6), 56.8 (-OCH_3_), 56.5 (-OCH_3_), 39.8 (C-8), 27.7 (C-11), 19.4 (C-9, 10). MS ESI-(−) *m*/*z* = 451.0/453.2 [M − H]^−^ for C_21_H_25_BrO_6_.

### 3.4. Synthesis of Compound ***6***

A suspension of compound **3** (98 mg) and NaOH (22 mg) in H_2_O (7.5 mL) was stirred about 30–40 min until it became clear. To this solution was added TBAI (9.8 mg) and *p*-xylylene dibromide (144 mg) in toluene (4.5 mL) dropwise. After stirring at room temperature for 6 h, the reaction mixture was extracted with ethyl acetate (EtOAc) (3 × 20 mL). The combined EtOAc layer was concentrated under reduced pressure to afford a residue, which was purified by chromatography on silica gel using acetone:hexane (1:2) to give **6** (44 mg, yield 17.8%).

*2-Methyl-1-{2,4,6-trihydroxy-3-[4-(2,4,6-trihydroxy-3-isobutyryl-benzyl)-benzyl]-phenyl}-propan-1-one* (**6**). ^1^H-NMR (400 MHz, CD_3_COCD_3_) δ: 14.19 (s, OH), 7.17 (4H, s, H-13, 13′, 14, 14′), 6.10 (2H, s, H-4, 4′), 4.02 (2H, m, H-8, 8′), 3.85 (4H, s, H-11, 11′), 1.14 (12H, d, *J* = 6.8 Hz, H-9, 9′, 10, 10′). ^13^C-NMR (100 MHz, CD_3_COCD_3_) δ: 210.1 (C-7, 7′), 164.8 (C-1, 1′), 162.0 (C-5, 5′), 159.7 (C-3, 3′), 138.9 (C-12, 12′), 128.1 (C-13, 13′, 14, 14′), 107.3 (C-6, 6′), 103.4 (C-2, 2′), 94.3 (C-4, 4′), 38.7 (C-8, 8′), 28.5 (C-11, 11′), 18.8 (C-9, 9′, 10, 10′). MS ESI-(−) *m*/*z* = 493.2 [M − H]^−^ for C_28_H_30_O_8_.

### 3.5. Synthesis of Compound ***7***

To a solution of piperidine (2.55 g) in CH_2_Cl_2_ (100 mL) was added K_2_CO_3_ (5.46 g). After stirring at room temperature for 1 h, *p*-xylenedibromide (7.86 g) in CH_2_Cl_2_ (100 mL) was added dropwise, and the reaction continued for 3 h. The reaction mixture was filtered. The filtrate was concentrated under reduced pressure, and the residue was purified by chromatography on silica gel using acetone:hexane (1:7) to yield the intermediate 1-(4-(bromomethyl) benzyl)piperidine (2.0 g).

To a solution of compound **3** (196 mg) and NaOH (44 mg) in H_2_O (60 mL) was added the intermediate (707 mg) in MeOH (40 mL) dropwise. After stirring at room temperature for 4 h, the reaction mixture was extracted with EtOAc (3 × 80 mL) and concentrated under reduced pressure. The residue was purified by chromatography on silica gel using CHCl_3_:MeOH:acetone (10:1:1) to yield **7** (35 mg, yield 9.2%).

*2-Methyl-1-[2,4,6-trihydroxy-3-(4-piperidin-1-ylmethyl-benzyl)-phenyl]-propan-1-one* (**7**). ^1^H-NMR (400 MHz, C_5_D_5_N) δ: 7.74 (2H, d, *J* = 6.0 Hz, H-13, 13′), 7.68 (2H, d, *J* = 6.0 Hz, H-14, 14′), 6.61 (1H, s, H-4), 4.37 (2H, s, H-11), 4.35 (1H, m, H-8), 4.23 (2H, s, H-16), 2.97 (4H, m, H-17, 17′), 1.77 (4H, m, H-18, 18′), 1.29 (6H, d, *J* = 6.7 Hz, H-9, 10), 1.28 (2H, m, H-19). ^13^C-NMR (100 MHz, C_5_D_5_N) δ: 211.2 (C-7), 166.4 (C-1), 164.6 (C-3), 162.5 (C-5), 145.2 (C-12), 132.16 (C-14, 14′), 130.3 (C-13, 13′), 127.89 (C-15), 107.5 (C-6), 104.8 (C-2), 95.9 (C-4), 61.0 (C-16), 52.99 (C-17, 17′), 39.7 (C-8), 29.2 (C-11), 23.62 (C-18, 18′), 22.78 (C-19), 20.3 (C-9, 10). HRTOF-MS ESI-(+) *m*/*z* = 384.2170 [M + H]^+^ for C_23_H_30_NO_4_ (cal. 384.2176).

^1^H-NMR for the isomer of **7** (400 MHz, C_5_D_5_N) δ: 7.78 (2H, d, *J* = 5.8 Hz, H-13, 13′), 7.63 (2H, d, *J* = 5.8 Hz, H-14, 14′), 6.61 (1H, s, H-4), 4.97 (2H, s, H-16), 4.37 (2H, s, H-11), 4.35 (1H, m, H-8), 2.97 (4H, m, H-17, 17′), 1.77 (4H, m, H-18, 18′), 1.36 (2H, m, H-19), 1.29 (6H, d, *J* = 6.7 Hz, H-9, 10). ^13^C-NMR for the isomer of **7** (100 MHz, C_5_D_5_N) δ: 211.2 (C-7), 166.4 (C-1), 164.6 (C-3), 162.5 (C-5), 145.2 (C-12), 132.23 (C-14, 14′), 130.3 (C-13, 13′), 128.04 (C-15), 107.5 (C-6), 104.8 (C-2), 95.9 (C-4), 64.3 (C-16), 53.25 (C-17, 17′), 39.7 (C-8), 29.2 (C-11), 23.84 (C-18, 18′), 23.01 (C-19), 20.3 (C-9, 10).

### 3.6. Assay for iNOS Inhibition

The assay was performed in mouse macrophages (RAW264.7, obtained from ATCC)) cultured in phenol red free RPMI medium supplemented with 10% bovine calf serum and 100 U/mL penicillin G sodium, and 100 μg/mL streptomycin at 37 °C in an atmosphere of 5% CO_2_ and 95% humidity. Cells were seeded in 96-well plates (50,000 cells/well) and incubated for 24 h for a confluency of 75% or more. Test samples diluted in serum free medium were added, and after 30 min of incubation, LPS (5 μg/mL) was added and cells were further incubated for 24 h. The concentration of nitric oxide (NO) was determined by measuring the level of nitrite in the cell culture supernatant by using Griess reagent. Percent inhibition of nitrite production by the test compound was calculated in comparison to the vehicle control. IC_50_ values were obtained from concentration response curves. Parthenolide was used as a positive control [25].

### 3.7. Assay for NF-κB Inhibition

The assay was performed in human chondrosarcoma cells (SW1353, obtained from American Type Culture Collection (ATCC), Manassas, VA, USA). Cells were cultured in 1:1 mixture of DMEM/F12 supplemented with 10% fetal bovine serum (FBS), 100 U/mL penicillin G sodium, and 100 μg/mL streptomycin at 37 °C in an atmosphere of 5% CO_2_ and 95% humidity. Cells (1.2 × 10^7^) were washed once in an antibiotic and FBS-free DMEM/F12, and then resuspended in 500 μL of antibiotic-free DMEM/F12 containing 2.5% FBS. NF-κB luciferase plasmid construct was added to the cell suspension at a concentration of 50 μg/mL and incubated for 5 min at room temperature. The cells were electroporated at 160 V and one 70-ms pulse using BTX disposable cuvettes model 640 (4-mm gap) in a BTX Electro Square Porator T 820 (BTX I, San Diego, CA, USA). The transfected cells were plated to the wells of 96-well plates at a density of 1.25 × 10^5^ cells per well. After 24 h, cells were treated with different concentrations of test compound for 30 min before the addition of PMA (70 ng/mL) and incubated for 8 h. Luciferase activity was measured using the Luciferase Assay kit (Promega). Light output was detected on a SpectraMax plate reader. Percent inhibition of luciferase activity was calculated as compared to vehicle control, and IC_50_ values were obtained from concentration response curves. Parthenolide was used as positive control [25].

### 3.8. Cytotoxicity Assay

Cytotoxicity was determined against the mammalian cell line Vero (African green monkey kidney fibroblast) which was obtained from ATCC. The detailed assay procedure has been described previously [26]. In brief, cells were seeded in 96-well plates (10,000 cells /well), and after 24 h of incubation, they were treated with various dilutions of test samples for 48 h. The cell viability was determined by tetrazolium dye WST-8. Doxorubicin was included as drug control.

## Figures and Tables

**Table 1:** Anti-inflammatory activity of synthetic acylphloroglucinols.

Compound	iNOS Inhibition	NF-κB Inhibition
	(IC_50_, µM)	(IC_50_, µM)
**2**	19.0 ± 1.4	34.0 ± 8.5
**3**	NA	90.0 ± 14.1
**4**	19.5 ± 0.7	37.5 ± 3.5
**5**	50.0 ± 0	29.0 ± 15.5
**6**	19.0 ± 1.4	85.0 ± 21.2
**7**	49.0 ± 1.4	NA
Parthenolide	2.5 ± 0.2	20.5 ± 2.9

NA, not active at 50 µM for iNOS and 100 µM for NF-κB.

## Supplementary Materials

The following are available online. Supplementary Materials, including MS, HRMS, 1D and 2D NMR spectra for the synthetic compounds, are available online.
