# Supplementary material for: Synthesis and Anti-Inflammatory Activities of Phloroglucinol-Based Derivatives
Source: Molecules. 2018 Dec 7;23(12):3232. doi: 10.3390/molecules23123232 (PMC6321231; doi:10.3390/molecules23123232)

Supplementary Materials

Synthesis and Anti-inflammatory Activities of Phloroglucinol-Based Derivatives

Ning Li[1,2], Shabana I. Khan[1,3], Shi Qiu[1,3] and Xing-Cong Li[1,3,*]

[1] Nation Center for Natural Products Research, Research Institute of Pharmaceutical Sciences, School of Pharmacy, The University of Mississippi, Mississippi 38677, United States

[2] School of Pharmacy, Anhui Medical University, Hefei 230032, P.R. China; ahmulining@163.com

[3]Department of Biomolecular Sciences, School of Pharmacy, The University of Mississippi, University, Mississippi 38677, United States

Table of Contents

1. ^{1}H-NMR and ^{13}C-NMR spectra of compound **2** S2

2. ^{1}H-NMR and ^{13}C-NMR spectra of compound **3** S3

3. ^{1}H-NMR and ^{13}C-NMR spectra of compounds **4** S4

4. ^{1}H-NMR and ^{13}C-NMR spectra of compound **5** S5

5. ^{1}H-NMR and ^{13}C-NMR spectra of compound **6** S6

6. ^{1}H-NMR and ^{13}C-NMR spectra of compound **7** in C_5D_5N S7

7. H-H COSY and HSQC spectra of compound **7** in C_5D_5N S8

8. HMBC in C_5D_5N and TOF MS ES$^+$ spectrum of compound **7** S9

9. ^{1}H-NMR and ^{13}C-NMR spectra of compound **7** in CD_3OD..S10

10. ^{1}H-NMR spectrum of compound **7** in $CDCl_3$ and ^{1}H-NMR spectra in different solvents of compound **7** S11

11. MS ESI (-) spectra of compounds **4-6** S12

S2. ^{1}H-NMR spectrum of compound **2** (400 MHz, CD_3COCD_3)

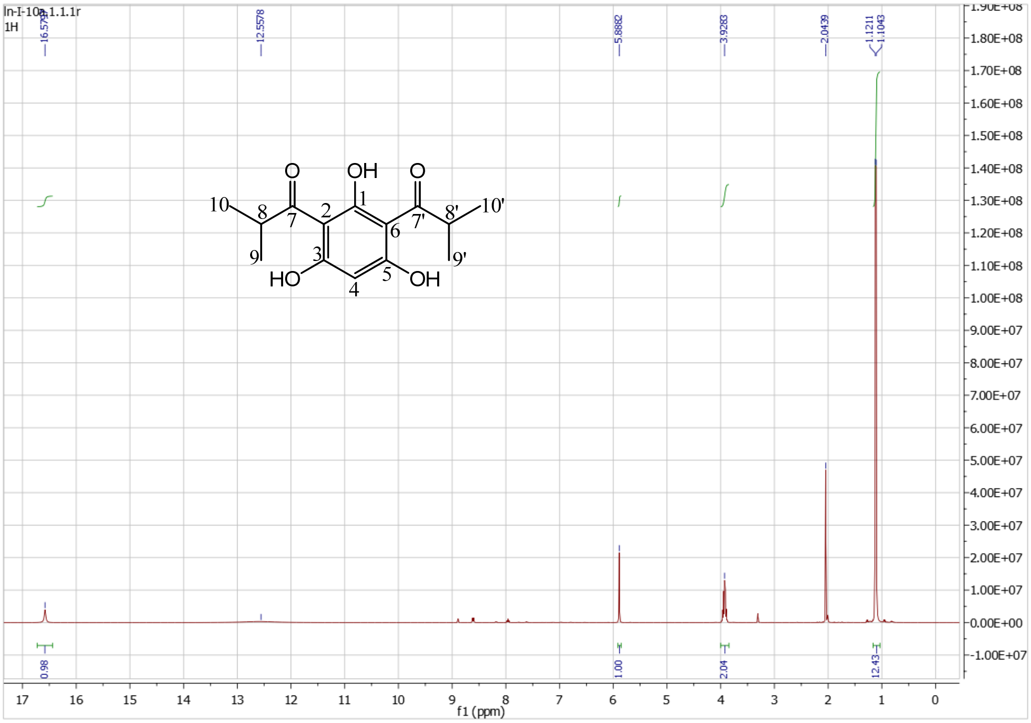

S2. ^{13}C-NMR spectra of compound **2** (100 MHz, CD_3COCD_3)

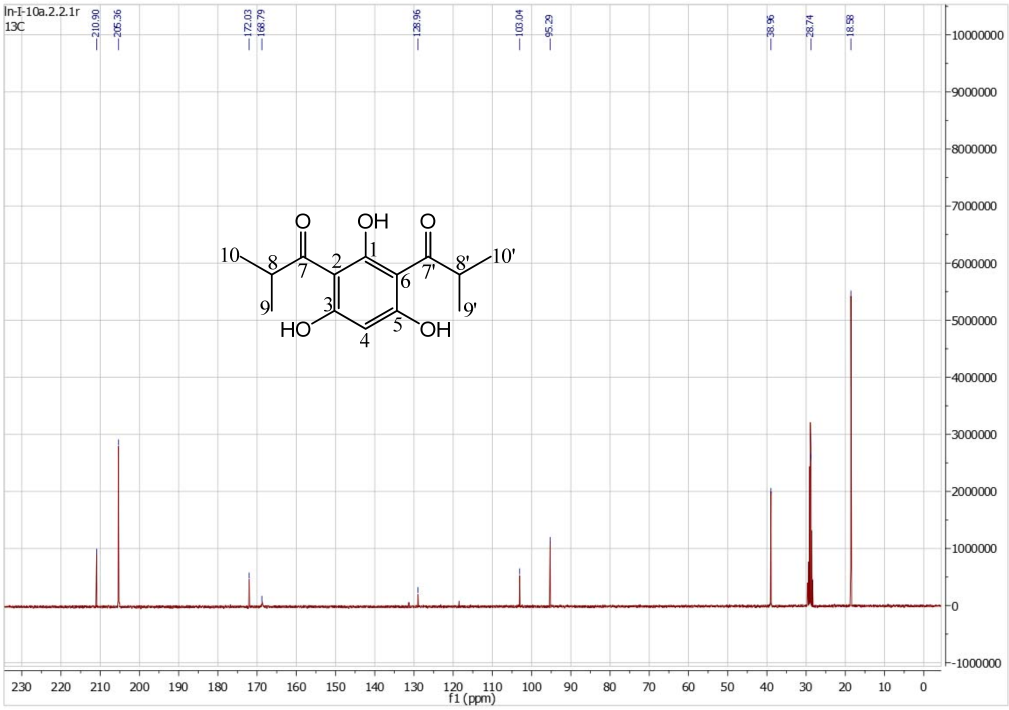

S3. ^{1}H-NMR spectrum of compound **3** (400 MHz, CD_3COCD_3)

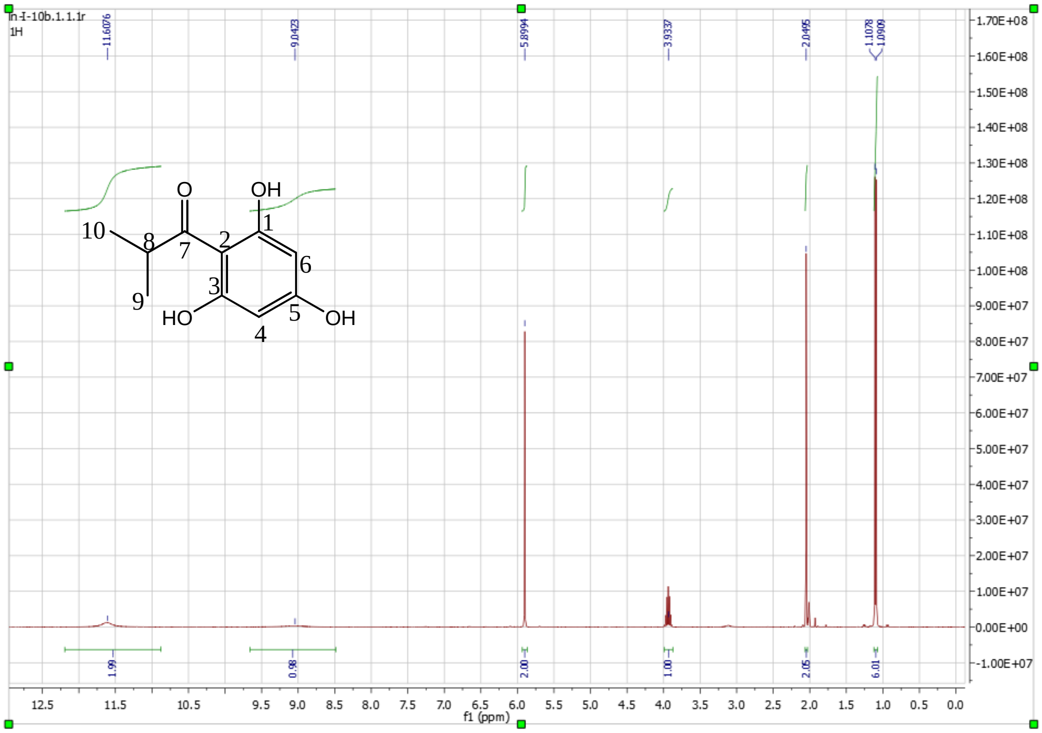

S3. ^{13}C-NMR spectra of compound **3** (100 MHz, CD_3COCD_3)

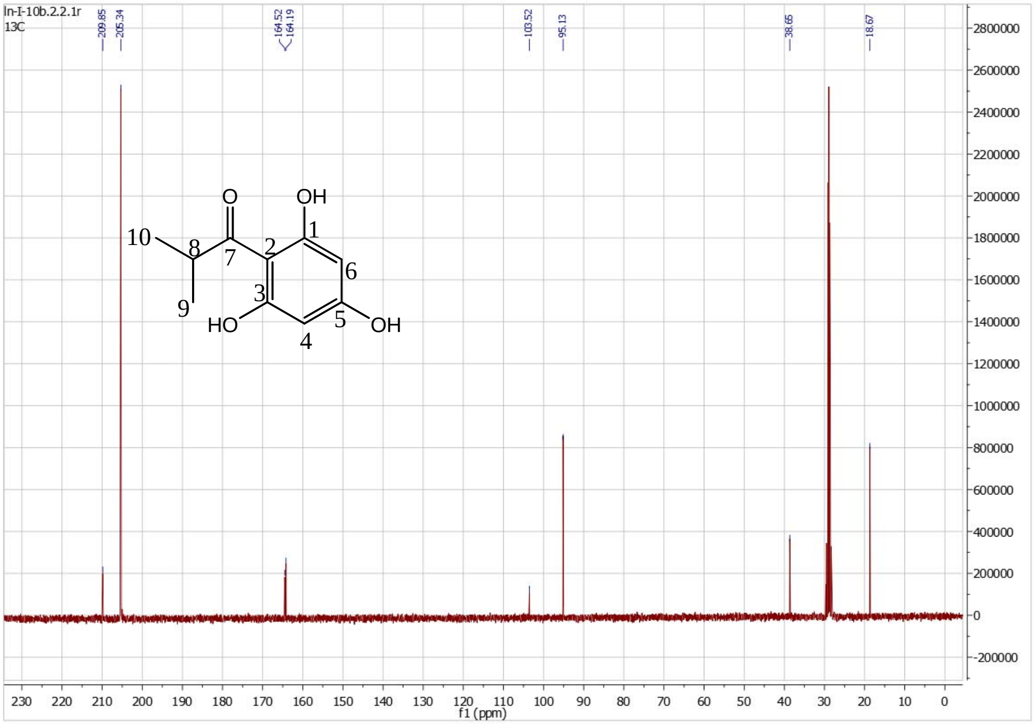

S4. ^{1}H-NMR spectrum of compound **4** (400 MHz, CD_3COCD_3)

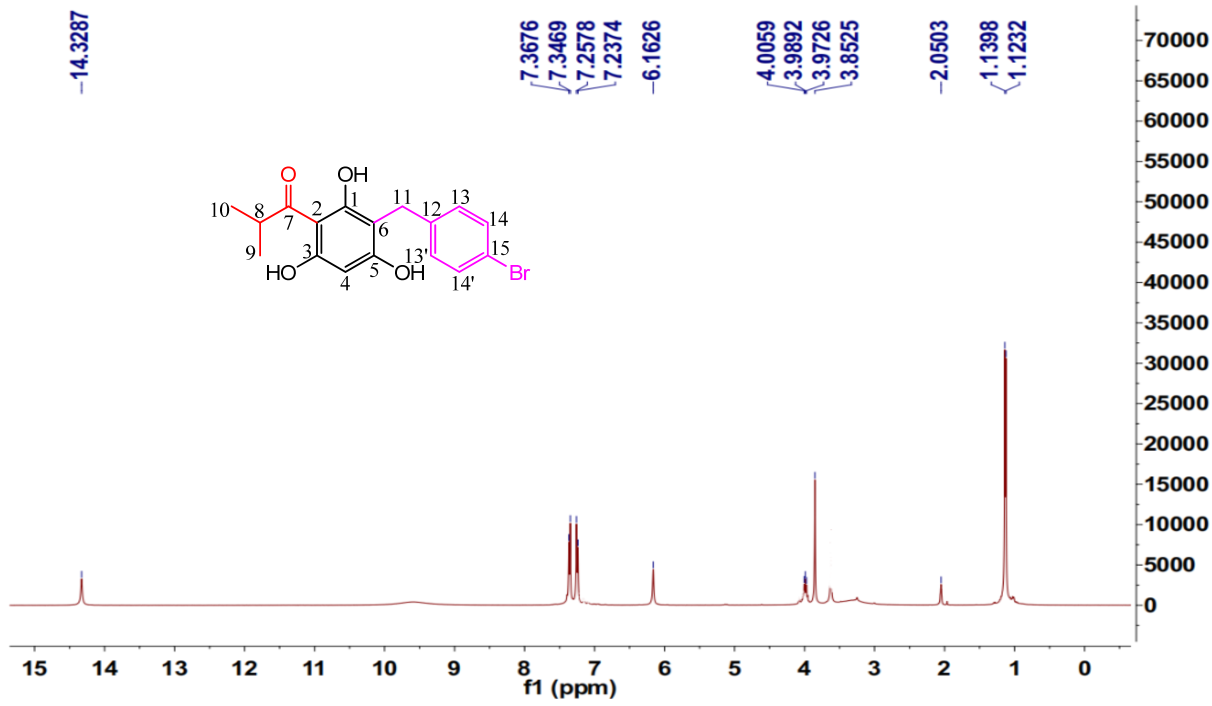

S4. ^{13}C-NMR spectrum of compound **4** (100 MHz, CD_3COCD_3)

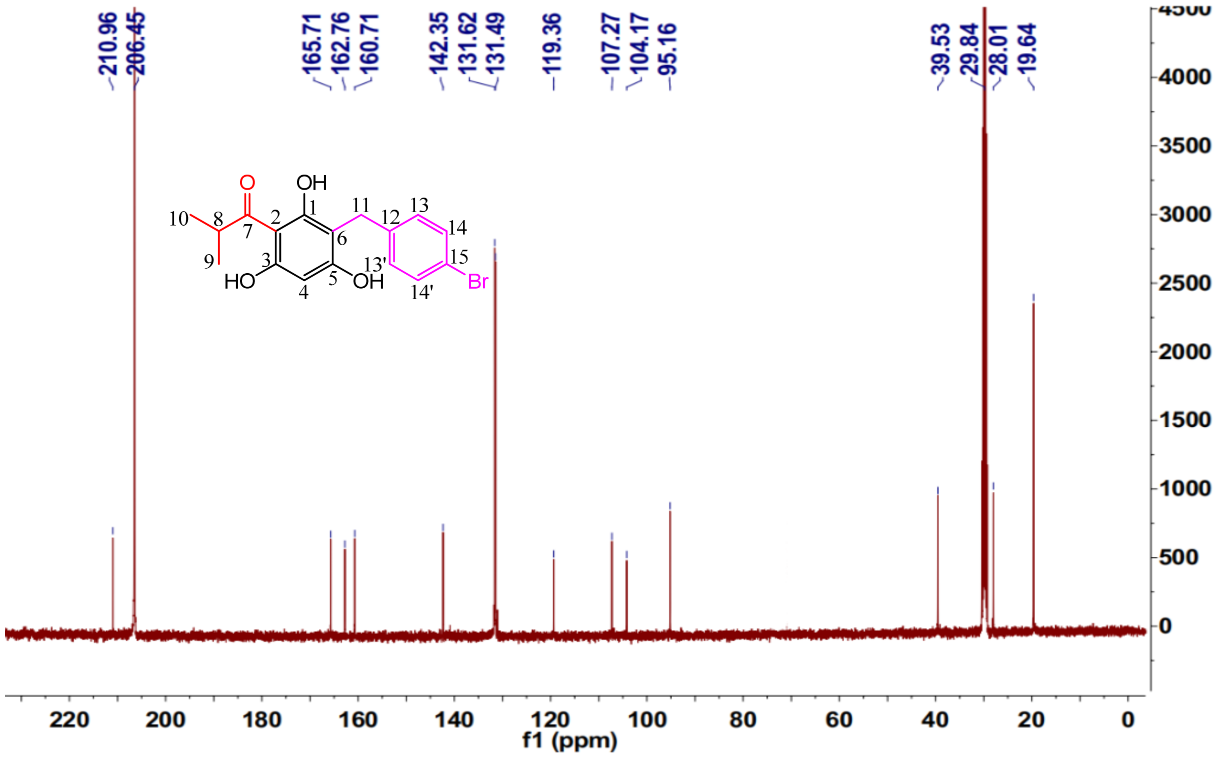

S5. ^{1}H-NMR spectrum of compound **5** (400 MHz, $CDCl_3$)

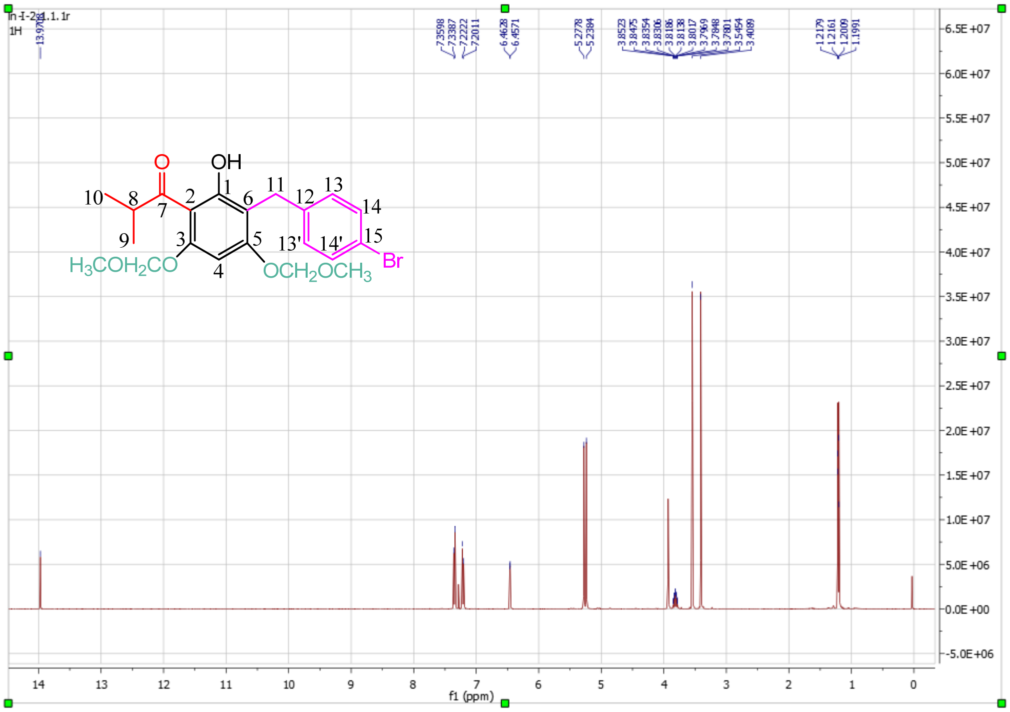

S5. ^{13}C-NMR spectrum of compound **5** (100 MHz, $CDCl_3$)

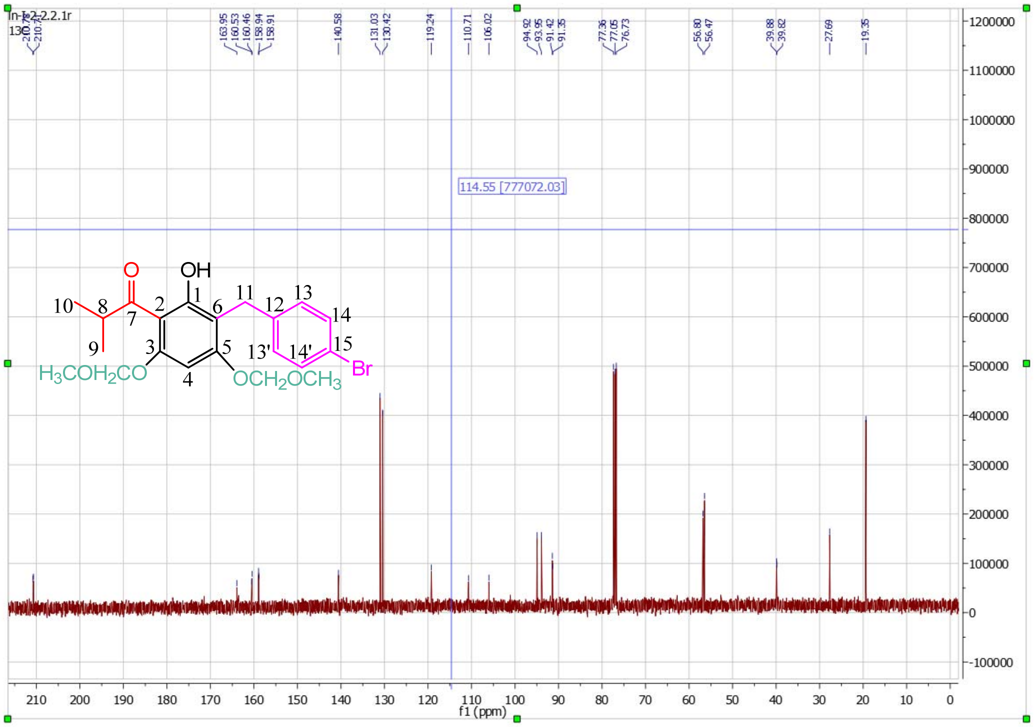

S6. ^{1}H-NMR spectrum of compound **6** (400 MHz, CD_3COCD_3)

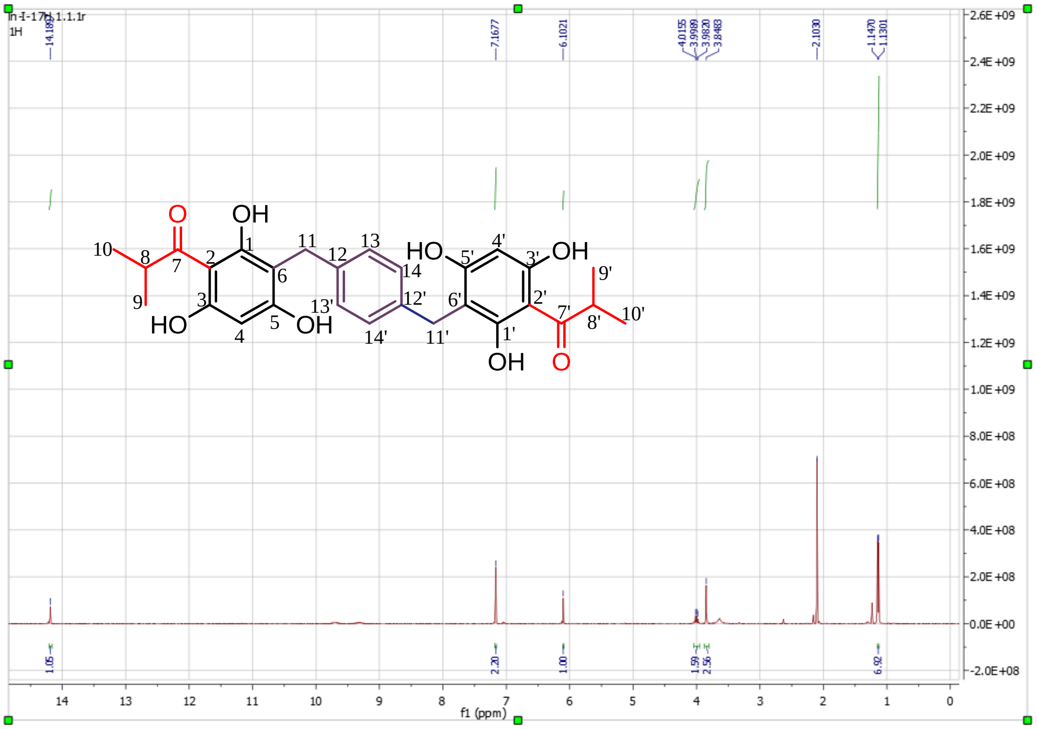

S6. ^{13}C-NMR spectrum of compound **6** (100 MHz, CD_3COCD_3)

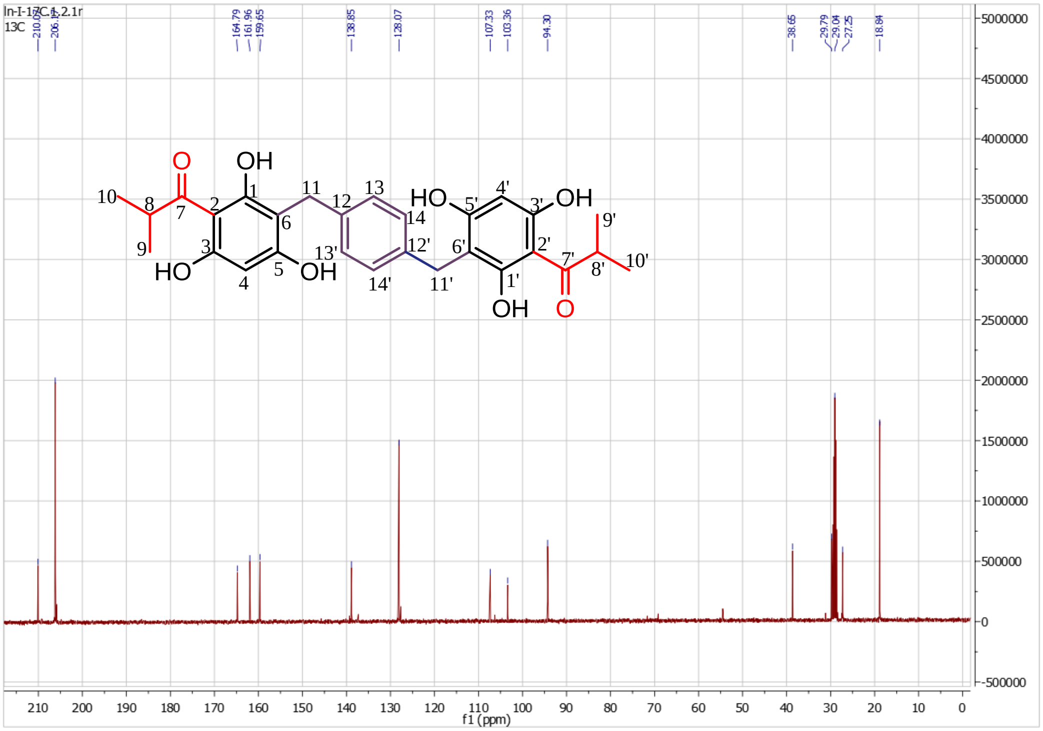

S7. ^{1}H-NMR spectrum of compound **7** (400 MHz, C_5D_5N)

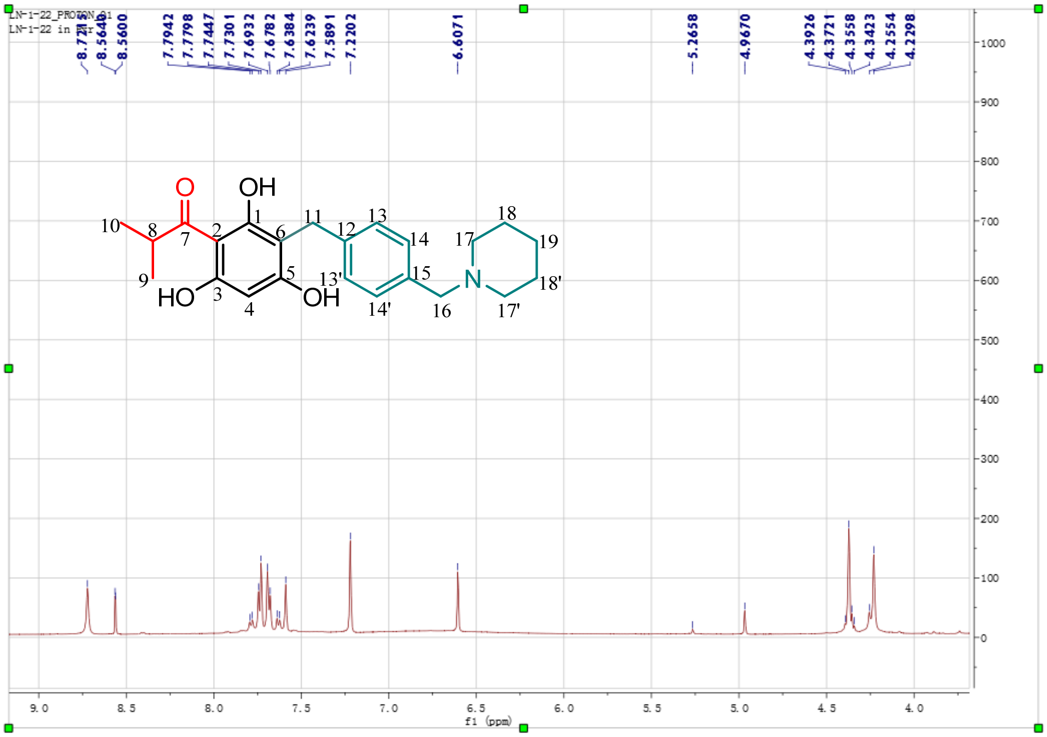

S7. ^{13}C-NMR spectrum of compound **7** (100 MHz, C_5D_5N)

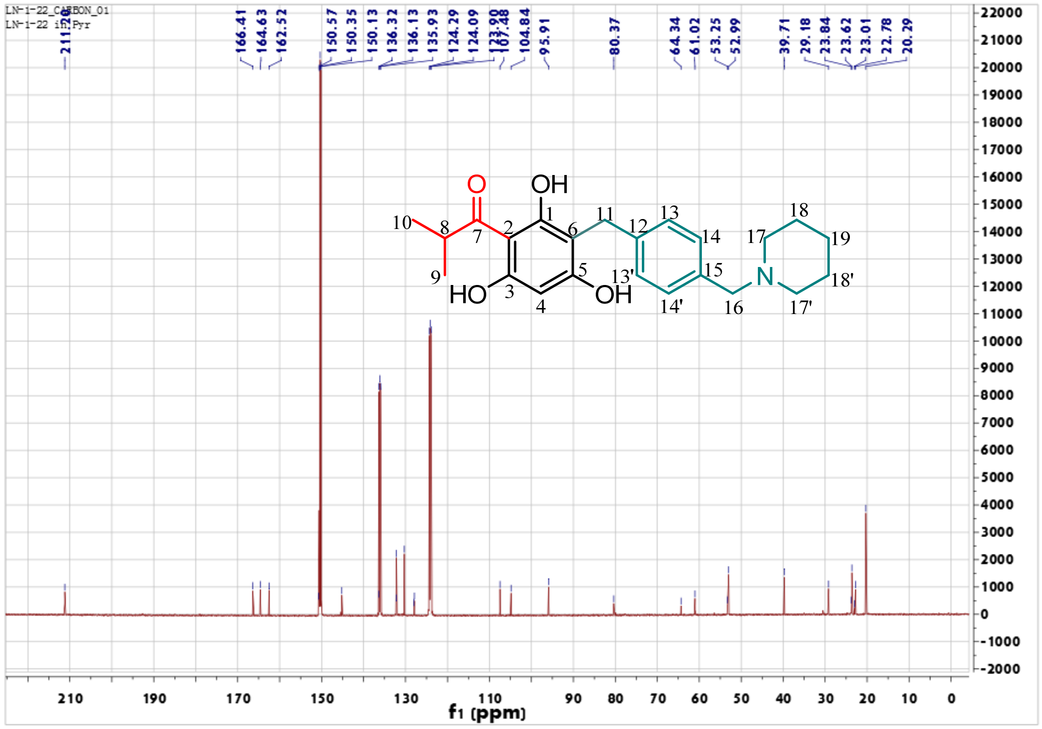

S8. H-H COSY spectrum of compound **7** in C_5D_5N

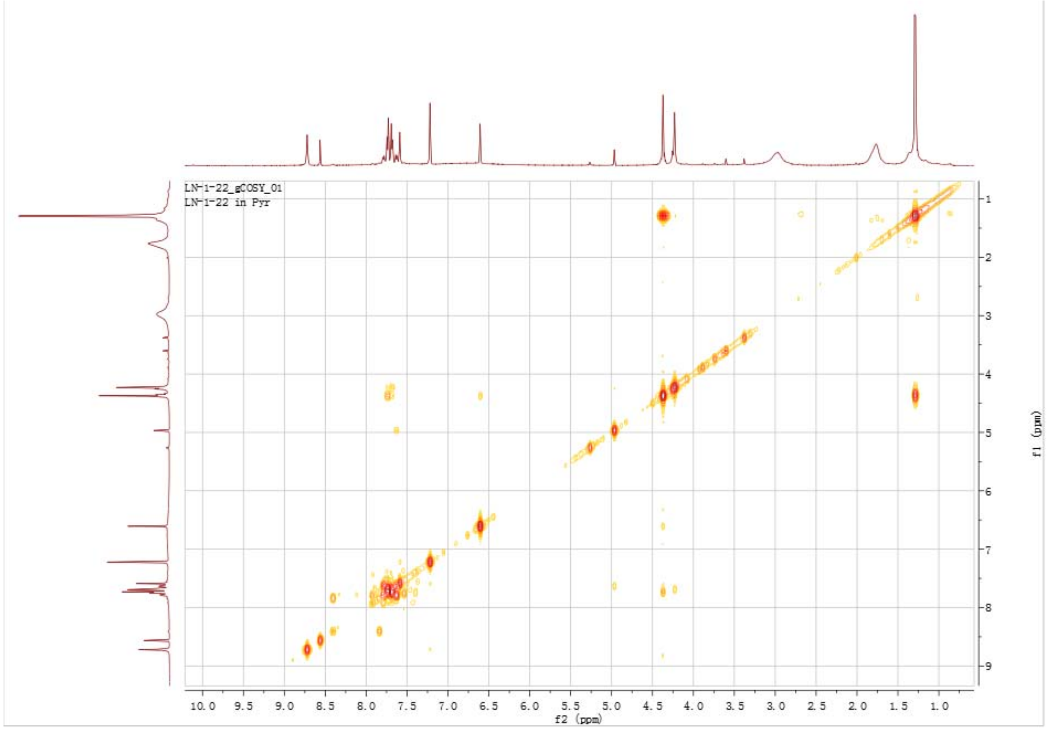

S8. HSQC spectrum of compound **7** in C_5D_5N

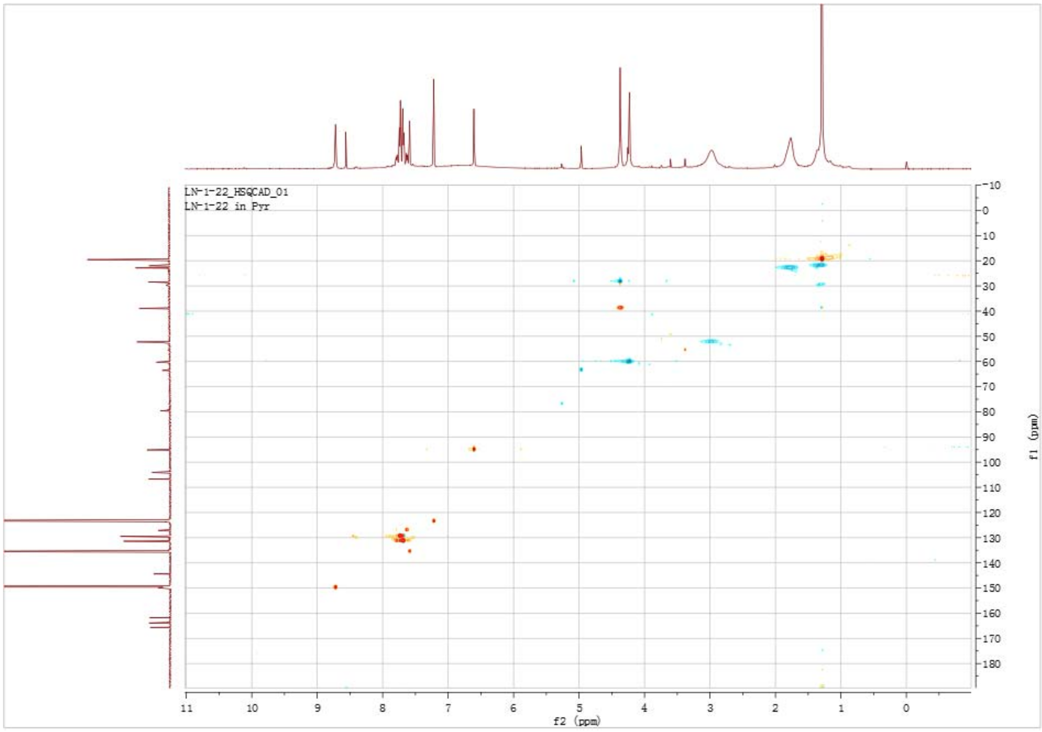

S9. HMBC spectrum of compound **7** in C_5D_5N

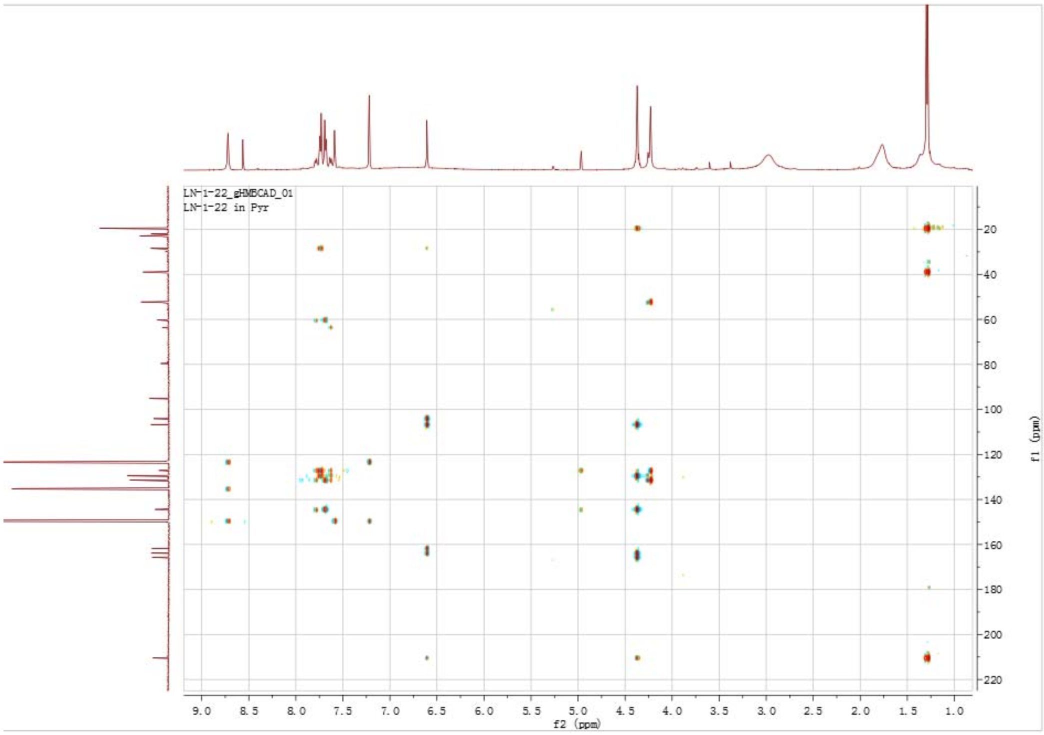

S9. TOF MS ES^+ spectrum of compound **7**

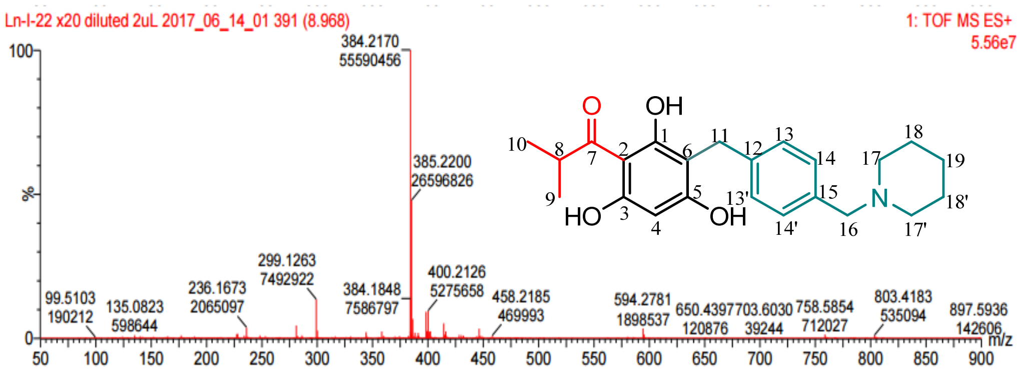

S10. ^{1}H-NMR spectrum of compound **7** (400 MHz, CD_3OD)

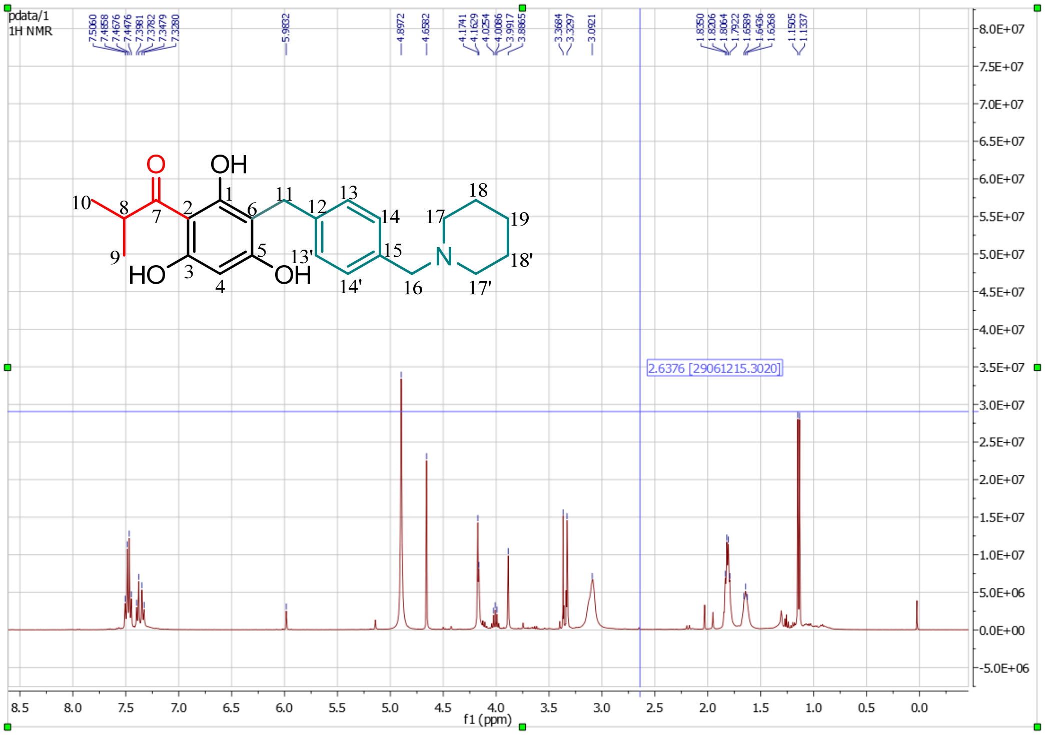

S10. ^{13}C-NMR spectrum of compound **7** (100 MHz, CD_3OD)

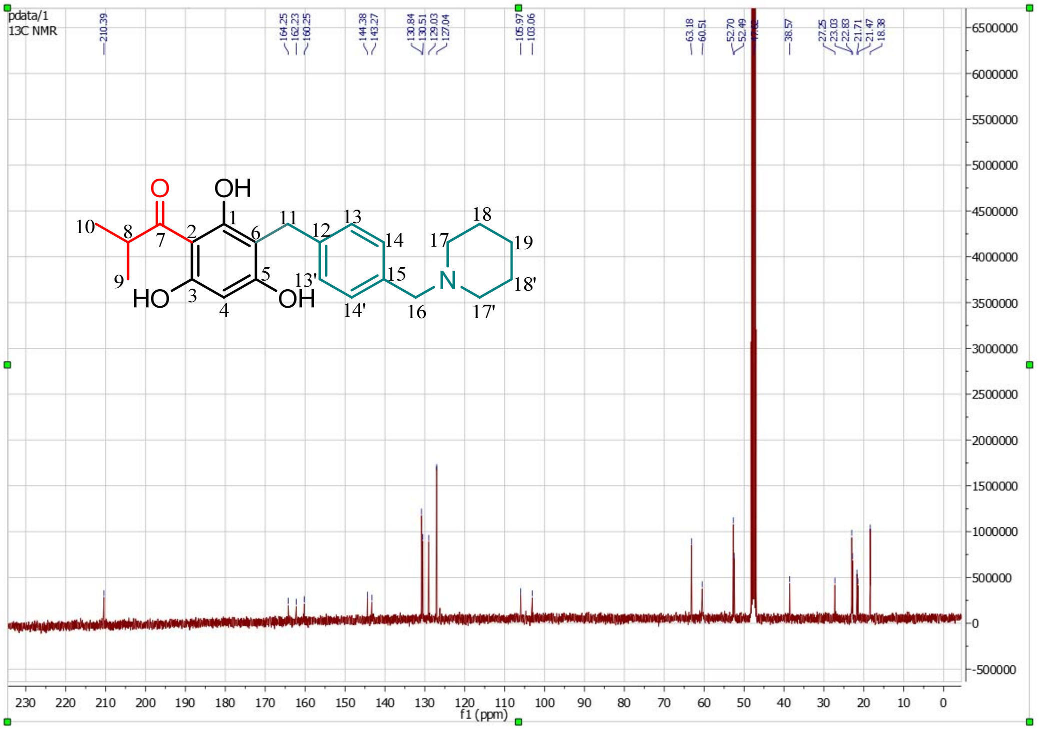

S11. ^{1}H-NMR spectrum of compound **7** (400 MHz, $CDCl_3$)

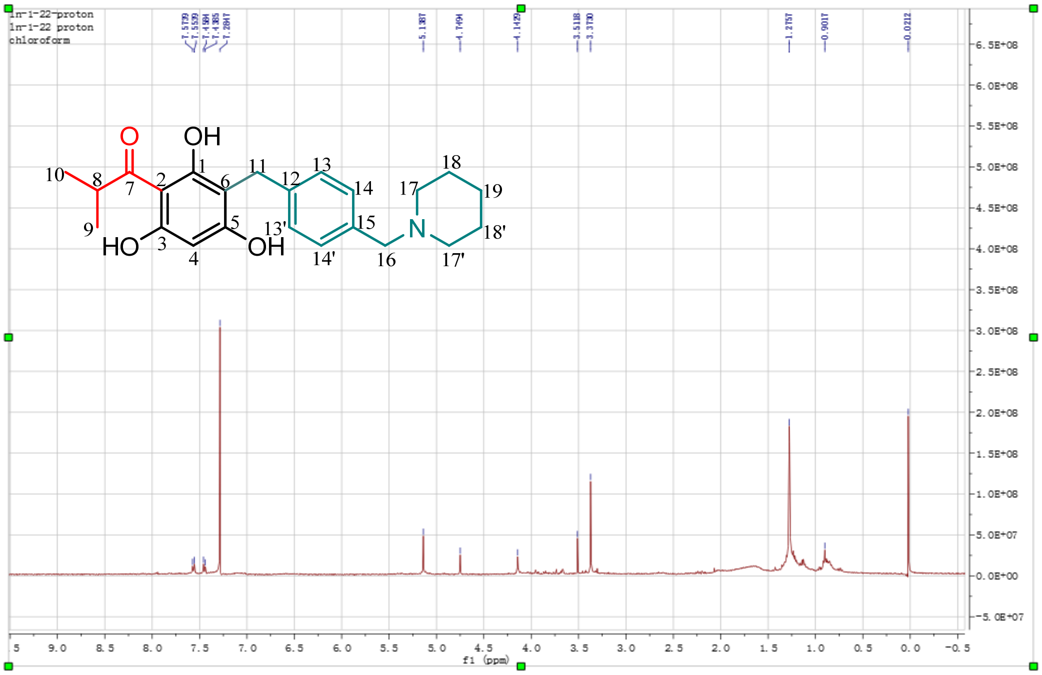

S11. ^{1}H-NMR spectra of compound **7** in different solvents

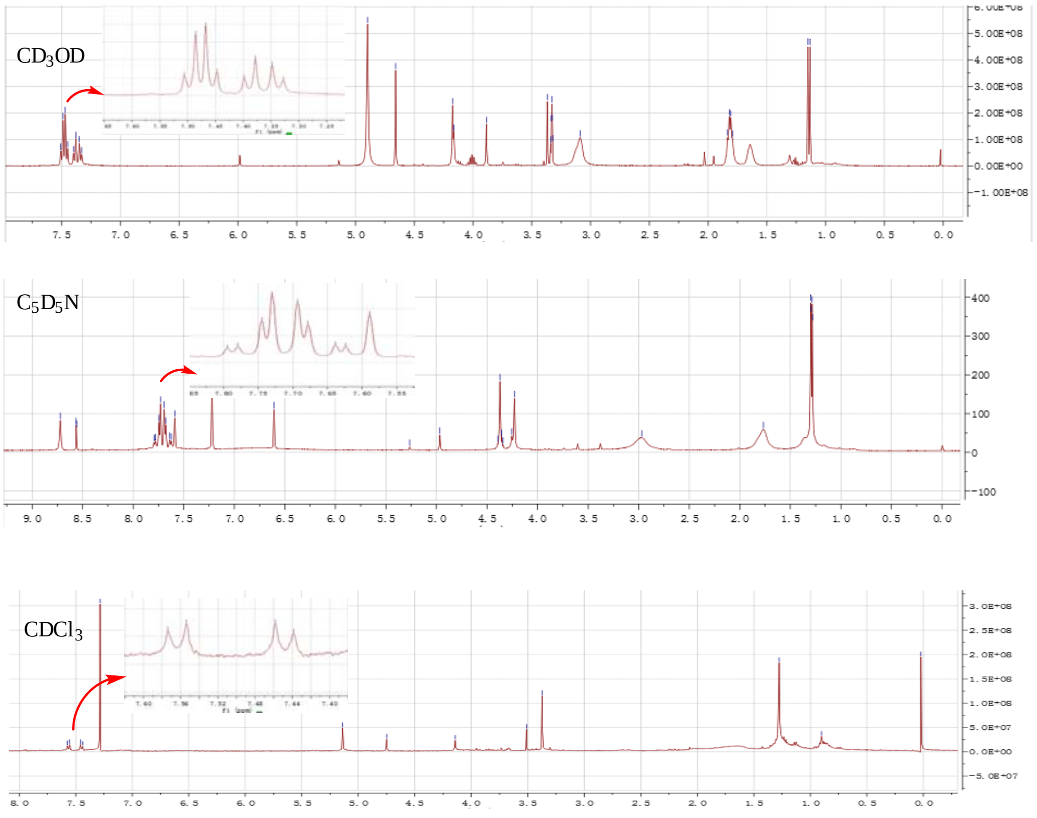

S12. MS ESI (-) spectra of compounds **4-6**

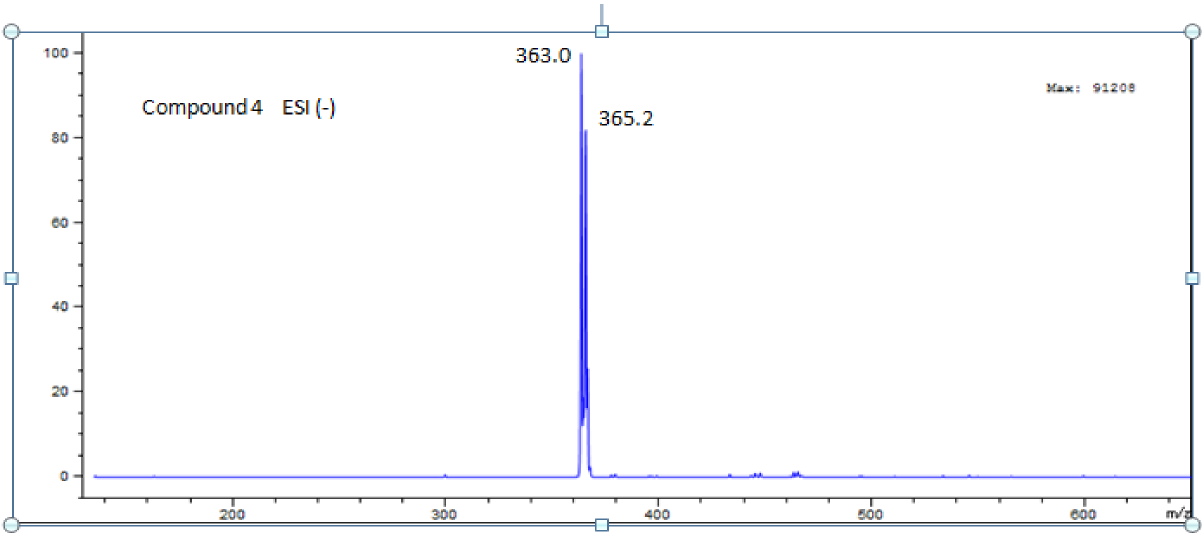

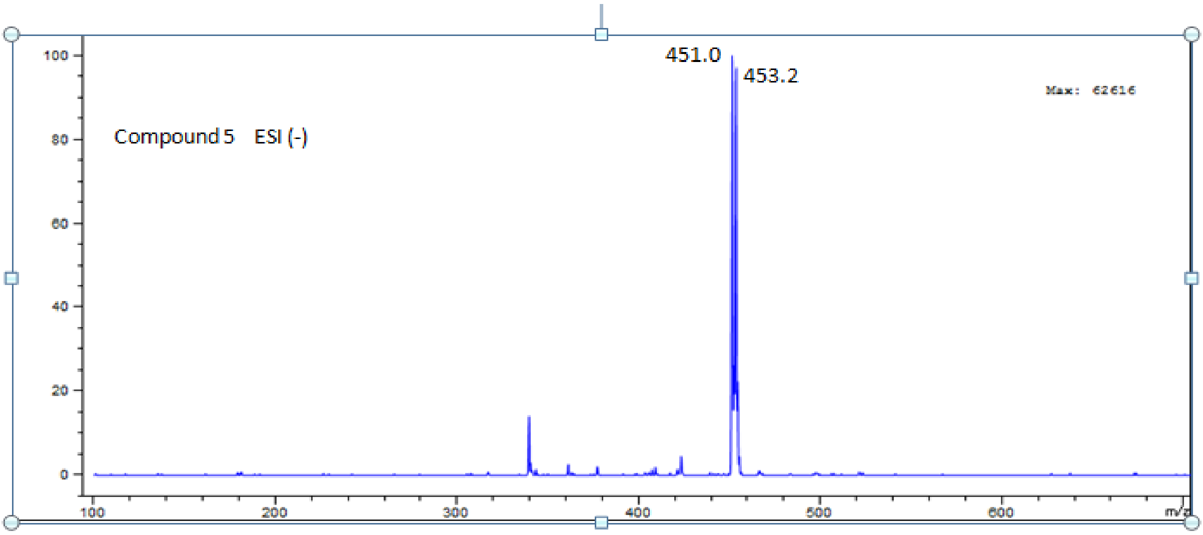

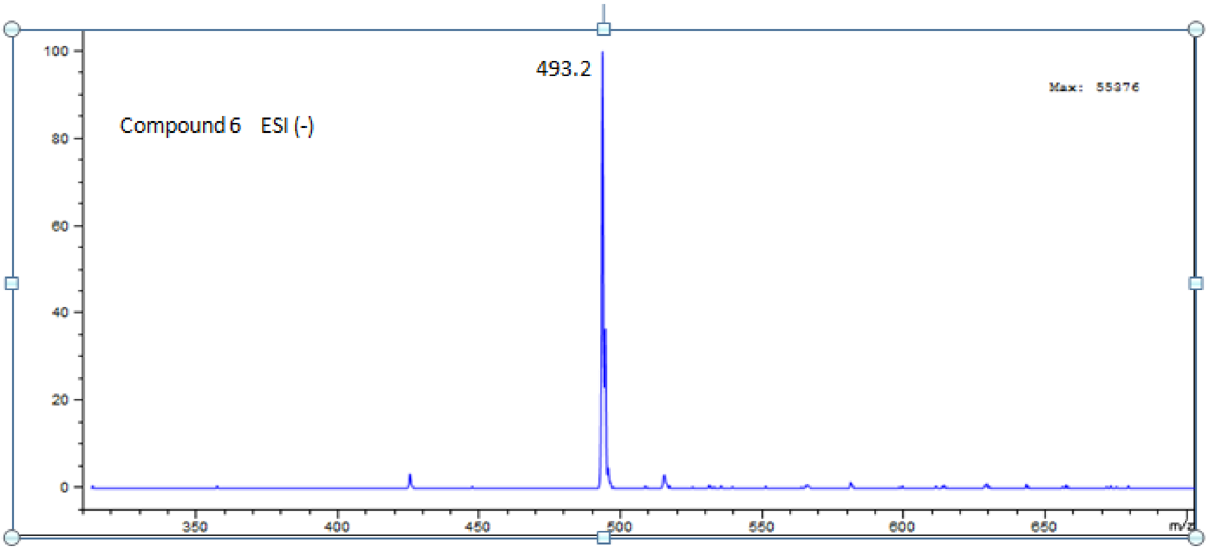

Supplement: Supplementary file 1 [file molecules-23-03232-s001.pdf]
